# Paper-Based Competitive Immunochromatography Coupled with an Enzyme-Modified Electrode to Enable the Wireless Monitoring and Electrochemical Sensing of Cotinine in Urine

**DOI:** 10.3390/s21051659

**Published:** 2021-02-28

**Authors:** Nutcha Larpant, Pramod K. Kalambate, Tautgirdas Ruzgas, Wanida Laiwattanapaisal

**Affiliations:** 1 Biosensors and Bioanalytical Technology for Cells and Innovative Testing Device Research Unit, Chulalongkorn University, Bangkok 10330, Thailand; Nutchalp.l@gmail.com (N.L.); Pramodkalambate1@gmail.com (P.K.K.); 2 Department of Biomedical Science, Faculty of Health and Society, Malmö University, SE-205 06 Malmö, Sweden; Tautgirdas.ruzgas@mau.se; 3 Biofilms—Research Center for Biointerfaces, Malmö University, SE-205 06 Malmö, Sweden; 4 Department of Clinical Chemistry, Faculty of Allied Health Sciences, Chulalongkorn University, Bangkok 10330, Thailand

**Keywords:** cotinine, immunochromatography, wireless biosensor, nanomaterials

## Abstract

This paper proposes a combined strategy of using paper-based competitive immunochromatography and a near field communication (NFC) tag for wireless cotinine determination. The glucose oxidase labeled cotinine antibody specifically binds free cotinine in a sample, whereas the unoccupied antibody attached to BSA-cotinine at the test line on a lateral flow strip. The glucose oxidase on the strip and an assistant pad in the presence of glucose generated H_2_O_2_ and imposed the Ag oxidation on the modified electrode. This enabled monitoring of immunoreaction by either electrochemical measurement or wireless detection. Wireless sensing was realized for cotinine in the range of 100–1000 ng/mL (R^2^ = 0.96) in PBS medium. Undiluted urine samples from non-smokers exhibited an Ag-oxidation rate three times higher than the smoker’s urine samples. For 1:8 diluted urine samples (smokers), the proposed paper-based competitive immunochromatography coupled with an enzyme-modified electrode differentiated positive and negative samples and exhibited cotinine discrimination at levels higher than 12 ng/mL. This novel sensing platform can potentially be combined with a smartphone as a reader unit.

## 1. Introduction

A wide variety of well-established technologies applied to in-vitro diagnostic testing have been elucidated by laboratories throughout the world. However, limited budgets and small-scale infrastructure of hospitals and healthcare management in remote areas are vital driving factors of the need for point-of-care testing (POCT) platforms [1]. Telemedicine applications are considered promising tools for the self-monitoring of health status that more actively facilitate care at patients’ homes [2]. The growth of emerging technologies in POCT devices is recently focused on diverse strategies, including novel platforms and assay formats, long-term stability, and storage of reagents [3]. Various innovations have been deployed to meet the requirement of POCT objectives, for instance, paper-based analytical devices [4], cell phone-based technologies [5], and lab-on-a-chip platforms [6]. The key features of patient-centered testing should be user-friendliness, minimized reagent and sample volume, robustness in sample processing, low cost, portability, and low turn-around time [7,8]. However, there is room for improving the near-patient testing format, for example, the skills of users and the handling of the pre-analytical, analytical, and post-analytical errors [9,10]. Although the immunochromatography detection method is quick and easy, there can be confusion in interpretation which leads to post-analytical errors. Naked-eye detection may result in incorrect interpretation due to variation in color perceptions between users [11]. To overcome this obstacle in the conventional lateral flow test, other techniques have been coupled with the platform to assist in interpretation [12]. Smartphone-assisted interpretation, which depends on image processing by built-in cameras [13], is quite popular [14,15,16]. In the Internet of Things (IoT) era, near field communication (NFC) has been widely used for data communication between devices as it is a low-cost wireless data transmission technology. NFC is based on radio frequency identification standards (RFID) that operate at 13.56 MHz. This standard protocol provides high security, a simple operation process, suitability for real-time signal measurement, low battery consumption in passive mode, high speed, and non-contact data transmission [17,18]. These versatile properties have generated attempts to use NFC tags as a smartphone-compatible sensing element. Apart from applications in security, goods inventory, and manufacturing, NFC tags have been applied to and integrated into food safety monitoring [19], chemical treats [20], and medical sensor devices [21]. There has been an increase in the development of NFC coupled with healthcare-related biosensor platforms [22]. Improvements include the non-invasive monitoring of metabolites or biochemical status [23], environmental exposure [24], human motion [25], and pathogen detection [26].

Cotinine, a dominant metabolite of nicotine, is considered a reliable tobacco-smoke exposure index due to its inactive form and long half-life in biological samples [27]. Besides its widespread use in smoking-cessation clinics to assess tobacco-smoke exposure, this metabolite is evaluated before surgery [28,29] or used for occupational purposes [30]. A simple method with satisfactory sensitivity and specificity needs to be developed for cotinine assay. To date, various approaches have been developed for cotinine determination, and lateral flow immunochromatography is the most common commercially available POCT method for cotinine assessment. Because of its small molecule, cotinine detection is based on a competitive immunoassay; however, visual readout bias still exists. Self-reported smoking status to monitor smoke cessation is not reliable as most patients do not confess their tobacco use, leading to inaccurate medical assessments. Hence, combining a conventional lateral flow immunochromatography with RFID sensing could be attractive for end-users to obtain accurate, easily assessable, and real-time data acquisition [31]. With the increased use of cloud computing systems for telemedicine [32], this approach shows great promise.

**Figure 1:**
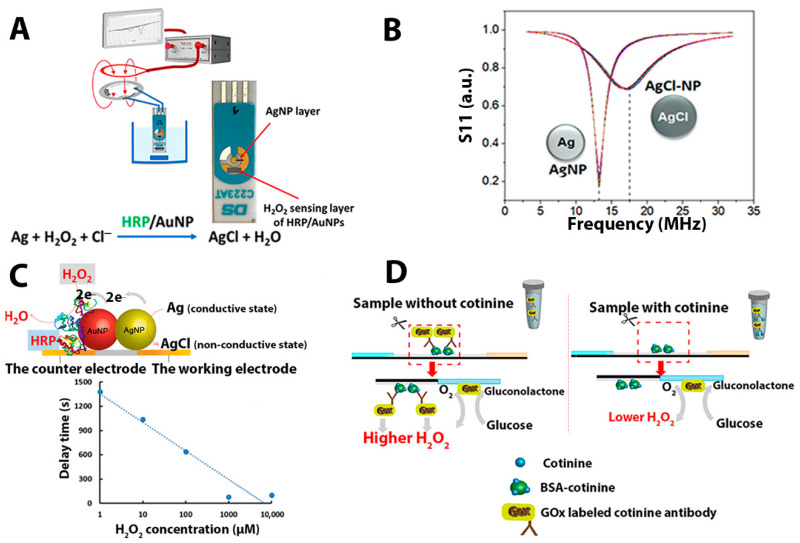
Wireless registration of H_2_O_2_ based on the direct electron transfer HRP/AuNP system. NFC-tag-based H_2_O_2_ biosensor configuration (**A**); the change of the reflection from conductive AgNP to non-conductive AgCl (S11, trace labeled with AgNP and AgCl-NP) (**B**); the reaction occurring on the surface of modified SPE combined with the cut nitrocellulose membrane and the shift-time H_2_O_2_ concentration dependence (**C**). Schematic illustration of the assay principle of cotinine detection by the proposed biosensor. The reaction occurring on the nitrocellulose membrane of the immunochromatography strip when the cotinine exists or absent in the sample (**D**).

Typically, oxidoreductases were exploited as the sensing part for diverse biosensor applications. This study exploits a previously described NFC-tag-based H_2_O_2_ biosensor configuration [33,34], where silver nanoparticles, as a redox reaction transducer into the wireless reaction registration mode, are deposited as a part of the NFC antenna [35]. To realize this construction, the antenna is broken and connected to working and counter electrodes of the screen-printed electrode (SPE). The electrodes are short-circuited by a layer of silver nanoparticles (AgNPs) (see Figure 1A). This tag-screen printed electrode connection has an electromagnetic reflection characteristic (S11) similar to the S11 of an intact NFC tag, with a resonance frequency close to 13.56 MHz. To sense H_2_O_2_, the layer of AgNPs is electronically coupled with horseradish peroxidase (HRP)-modified gold nanoparticles (AuNPs) where the presence of H_2_O_2_, converts AgNPs to AgCl. This breaks the antenna and changes the S11 characteristic, shifting the tag’s resonance frequency from 13.56 MHz to higher than 17 MHz (Figure 1B). Hence, the time needed to observe the resonance frequency shift (we call this a shift-time or delay time) is lower as the concentration of H_2_O_2_ increased (Figure 1C). This shift-time–concentration dependence illustrates the wireless biosensing principle. In this work, the idea of the wireless biosensor is extended to show that the wireless setup might be suitable for fabricating immunosensors. The immunosensing is demonstrated by coupling the wireless H_2_O_2_ biosensor to a simple paper-based lateral flow immunoassay. The integrated platform with an RFID-based biosensor was proposed to screen tobacco metabolites in a urine sample matrix that can be measured by a potentiostat and wirelessly monitored by a network analyzer or smartphone.

The assay principle is based on a competitive immunochromatography between free cotinine in the sample and BSA-cotinine on a lateral flow immunochromatography (Figure 1D). The test line contained pre-immobilized cotinine-BSA, which bound specifically to the cotinine antibody conjugated glucose oxidase (COT-Ab-GOx). The immunocomplex then is used to generate H_2_O_2_, which results in the transformation of the Ag to non-conductive AgCl by HRP catalyzing the breakdown of H_2_O_2_ on the AgNP/AuNP/HRP-modified screen-printed electrode (SPE). This conversion rate depends on the cotinine concentration in urine.

We have successfully demonstrated RFID cotinine sensing with real urine samples. Interference from ascorbic acid was greatly minimized using Nafion membrane filtration. This novel platform motivates the development of POCT devices to support decision-making processes, improve patient outcomes, and connect the data acquisition to other systems. The determination of other biomarkers or chemicals could also be addressed by this NFC-tag-based biosensor concept due to the universality of the biosensor platform. In particular, the recognition elements such as antibodies can be specifically adapted to other target analytes. Users could customize their biosensors, which electrochemical instruments or even smartphones can monitor.

## 2. Materials and Methods

### 2.1. Materials, Chemicals and Instruments

Phosphate buffer saline (PBS) tablets, silver nitrate (AgNO_3_), tri-sodium citrate, sodium chloride, potassium chloride, D-(+)-glucose, glucose oxidase from *Aspergillus niger* (lyophilized, powder, ~19.29 U/mg), horseradish peroxidase (type VI, lyophilized powder, ≥250 U/mg), L-ascorbic acid, and Nafion^®^ 117 solution were purchased from Sigma Aldrich (St. Louis, MO, USA). Glucose oxidase conjugation kit (ab102887) was imported from Abcam (Cambridge, UK). Cotinine-4 antibody (abx022798) and cotinine-4-BSA (abx080056) were obtained from Abbexa (Cambridge, UK). The cotinine antibody was conjugated with glucose oxidase by following the glucose oxidase conjugation kit protocol from Abcam. Gold screen printed electrodes (SPE) C223AT were from Dropsens, Llanera, Spain. RFID tags (NTAG216, with operation frequency 13.56 MHz) were obtained from Smart Digital Door Lock Ltd, Bangkok, Thailand. Gold nanoparticles (40 nm) were received from Kestral Bioscience (Bangkok, Thailand). Nitrocellulose membranes (Unisart CN 95, Sartorius, Göttingen, Germany), glass fiber membranes (GF 33) (Merck Millipore, Billerica, MA, USA), adsorbent pad grade 222 (Ahlstrom-Munksjö, Helsinki, Finland), and adhesive backing cards were used in the lateral flow assembly. Instant-view^®^ cotinine lateral flow tests were received from Alfa Scientific Designs, Inc. (Poway, CA, USA). Self-adhesive plastic film was acquired from Nitto Europe NV, Genk, Belgium. PTFE syringe filter 13 mm (0.22 µM) was purchased from Membrane Solutions (Auburn, WA, USA). All solutions were prepared by using deionized water purified by the Milli-Q system (Merck Millipore, Billerica, MA, USA) with a resistivity of 18.2 Ω cm.

The RFID measurements were conducted using DG8SAQ Vector Network Analyzer v3E from SDR-Kits (Melksham, UK). The electrochemical experiments were conducted using Autolab PGSTAT101 (Barendrecht, The Netherlands). Field emission scanning electron microscope (FESEM) and atomic force microscope (AFM) analysis were performed at the National Science and Technology Development Agency, Thailand. HITACHI SU8030 FESEM (Tokyo, Japan) and AFM 5500, HITACHI (Tokyo, Japan) were used to study the morphology of the modified electrode before and after Ag-oxidation. Gas chromatography-mass spectrophotometer (GC/MS), 7890A (GC) 5975C (MSD) Agilent Technologies (Los Angeles, CA, USA), was used to determine the quantity of cotinine and nicotine at the Toxicology Laboratory Service, Department of Pathology, Faculty of Medicine, Ramathibodi Hospital, Bangkok, Thailand.

### 2.2. Experimental Set up for Immunosensing of Cotinine

Two main components used in the immunosensing of cotinine are composed of competitive paper-based immunochromatography platform and AgNP /HRP/AuNP modified screen-printed electrode. The protocols set up for these crucial parts are list as following.

#### 2.2.1. Competitive Paper-Based Immunochromatography Platform Fabrication

**Figure 2:**
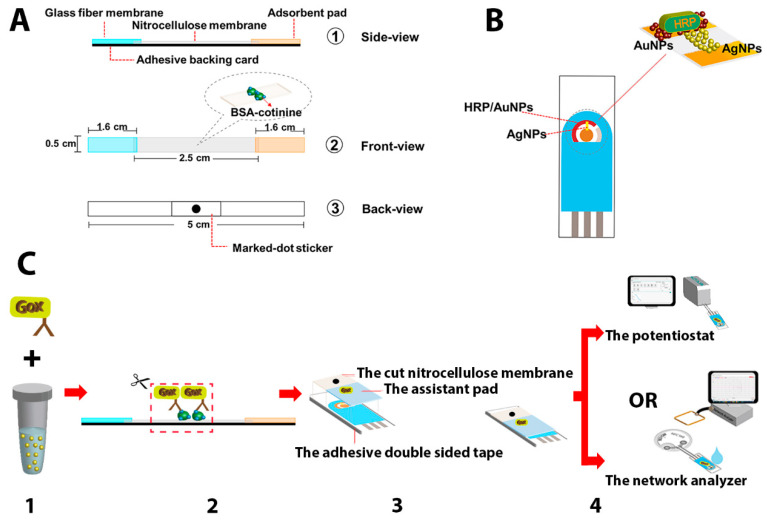
Experimental components for cotinine sensing. In-house paper-based immunochromatography for cotinine (**A**); the AgNP/HRP/AuNP -modified SPE (**B**). The analytical procedure of cotinine determination by the proposed biosensor (**C**). The procedure comprises four (1–4) steps; (1) Mix the sample with GOx labeled cotinine antibody. (2) Apply the mixed sample to paper-based lateral flow immunochromatography. (3) Attach the cut nitrocellulose membrane and the assistant pad to the electrode surface. (4) Monitor the network analyzer or the potentiostat after the substrate is added.

Five types of materials were exploited to fabricate the paper-based immunochromatography used in this study, including., glass fiber membrane, nitrocellulose membrane, backing card, absorbent pad, and printed marked dot sticker. The different sizes of materials were cut by a laser cutter (Cnmanlaser, model MAN-6090, Qingdao, China). The machine was set up with a CO_2_ laser power of 15 kW and a cutting speed of 30 mm/s. The following sizes for glass fiber membrane (5 mm × 16 mm), nitrocellulose membrane (5 mm × 25 mm), adsorbent pad (5 mm × 16 mm), and printed marked dot sticker (5 mm × 10 mm) were obtained. Before any surface modification, both the glass fiber and nitrocellulose membranes were pretreated by dropping 50 µL of PBS buffer (pH 7.2) and left to dry at room temperature. Then, 1 µL of 5 mg/mL BSA-cotinine was spotted on the nitrocellulose membrane and allowed to dry in ambient air for 1 h. Subsequently, all the membranes were assembled similarly to the conventional lateral flow immunochromatography stirp test, as demonstrated in Figure 2A.

#### 2.2.2. Preparation of AgNP/HRP/AuNP -Modified SPE

Silver nanoparticles (AgNPs) were synthesized following the method described by Li et al. [36]. The solution of synthesized AgNPs with the initial absorbance of 2.8 at 400 nm was then centrifuged to concentrate the particles. All the supernatant was removed, and the pellet of AgNPs was collected for further use. According to the manufacturer’s certificate, the absorbance of the AuNP solution at 523.5 nm was 1.188. It was also concentrated in the same manner as the AgNPs. For the electrode modification step, the SPE was first covered by a patterned self-adhesive plastic film to assist the AgNP immobilization into the area of a rectangular shape (2 mm width and 5 mm length). To connect the working and counter area of the SPE, 0.5 µL of concentrated AgNP was dropped into the hole of the adhesive film, then left to dry. After that, the self-adhesive plastic film was removed, creating AgNP layer modified SPE as shown in Figure 2B. This short-circuited modification connected the gap between the working and counter electrodes. After the patterned adhesive film was peeled off, the counter electrode was then covered with 2 µL of concentrated AuNPs onto the SPE surface. The modified SPE was then placed on a hot plate for drying at 65 °C and left to cool at room temperature before the subsequent experiment. Next, 5 µL of 2 mg/mL of HRP was dropped over the AuNP layer of the SPE to allow the enzyme to adsorb on the electrode surface at 4 °C overnight. The components and configuration of the AgNP/HRP/AuNP-modified SPE are demonstrated in Figure 2B. Both parts of the paper-based immunosensing platform and the AgNP/HRP/AuNP-modified electrode were subsequently used for cotinine determination with electrochemical (amperometric) and wireless (S11) sensing.

### 2.3. Analytical Procedure and Data Analysis

For cotinine assay by the proposed biosensor (see Figure 2C), 1 µL of anti-cotinine conjugated glucose oxidase was added to 500 µL of a urine sample and incubated for 15 min. Then 50 µL of sample/anti-cotinine conjugated glucose oxidase mixture was dropped onto the sample pad of the paper-based immunosensing platform. After a waiting period of 10 min, the absorbent pad was thoroughly wet due to the fluid wicking; then the strip was cut at a marked sticker area. The cut nitrocellulose membrane was attached to the AgNP/HRP/AuNP-modified SPE, in which the edges of the electrode and the membrane were attached with double-sided tape. To allow the immunosensing to occur on the electrode, the lateral flow nitrocellulose membrane’s front side was attached to the modified SPE surface. Another piece of a nitrocellulose membrane (size 10 mm × 10 mm) was dropped with 1 µL of 2 mg/mL GOx and used as an assistant pad to accelerate Ag oxidation on the electrode. The membrane was attached to the AgNP/HRP/AuNP-modified SPE, in which an edge of the membrane was 1-mm overlay combined to the cut nitrocellulose membrane from the previous step. To initiate the reaction, 100 µL of 5 mM glucose in citrate buffer (pH 6.0) was added to the gap between the electrode and the nitrocellulose membrane. In the first detection method, the amperometry-based measurement was conducted by applying 5 mV to the electrode, and the resulting current was recorded throughout the experiment. In the second approach, after the electrode was connected to the RFID tag, the frequency shift during the reaction was monitored and the data were collected every four seconds using a network analyzer instrument.

### 2.4. Interference Study

To investigate the interference effect on the proposed biosensor, 0.25 mM of ascorbic acid was spiked into the control urine without cotinine and with 1000 ng/mL of cotinine. Moreover, to reduce the sample matrix’s interference, the in-house modified syringe filter was used to demonstrate sample processing. Briefly, 0.5% Nafion solution was filtrated through a PTFE syringe filter, and the 0.5% Nafion/PTFE syringe filters were left in a hot air oven for overnight (65 °C). These in-house modified syringe filters were used to filtrate the urine control sample before assay. All the experiments were assessed by electrochemical measurements.

### 2.5. Real Sample Analysis

This project was approved by the Ethics Review Committee for Research Involving Human Research Subjects, Health Sciences Group, Chulalongkorn University (ECCU), Bangkok, Thailand (COA No.035/2019). Random midstream urine samples were collected from volunteers, smokers (*n* = 4), and non-smokers (*n* = 3), following the recommendations from the Clinical and Laboratory Standards Institute (CLSI), and directly used for analysis. All the urine samples were tested with our proposed biosensor and compared with commercial cotinine lateral flow strips. The same urine samples were also analyzed by the reference method for cotinine and nicotine by using a gas chromatography-mass spectrophotometer. The procedure for comparing with our proposed methods are described as follows.

For the commercial immunochromatographic assay, Instant-view^®^ cotinine lateral flow tests were used for providing detection of cotinine in human urine at a cut-off concentration of 200 ng/mL. Referring to the package insert, the test device is based on the principle of competitive binding containing mouse monoclonal anti-cotinine antibody-coupled particles and cotinine-protein conjugate. A goat antibody is employed in the control line system. Following to directions for use, 100 µL of urine sample was dropped to the specimen well. Wait until the sample reach to the end of adsorbent pad and read the result at 5 min and not be further than 10 min. In the interpretation of results, two appeared red lines at the test line and control line was considered as a negative result, whereas one appeared red line at control line was interpreted as a positive result. And if the control line fails to appear, there is considered as an invalid result.

For the reference method for the determination of cotinine and nicotine, the samples were analyzed using a GC/MS at the Toxicology Laboratory Service, Department of Pathology, Faculty of Medicine, Ramathibodi Hospital, Bangkok, Thailand. In the sample preparation, 1 mL of urine sample (treated with phosphate buffer, pH 6) was extracted with SPE. The analytes in the sample were eluted with 2 mL of dichloromethane/isopropyl alcohol/ammonium hydroxide (78/20/2 *v/v/v*). The extract was evaporated under a stream of N_2_ gas, and residue was constituted with 100 µL of ethyl acetate. For GC/MS operating conditions, the separation was achieved with HP-1 column (17 m × 0.20 mm, 0.11 µM) (Agilent Technologies, Los Angeles, CA, USA). The flow rate of helium was 1.0 mL/min. The operating parameters were as follows: transfer line temperature 280 °C; the column temperature was programmed from 80 to 320 °C (25 °C/min), then held for 1 min. For the MS mode, Selected ion monitoring was used to determine the target analyte as the following: *m/z* 84, 133, and 162 for nicotine and 98, 118, and 176 for cotinine.

## 3. Results and Discussions

In this work, we expand the application of wireless NFC-tag-based biosensors to the area of immunosensing. The main concept of an NFC-tag-based biosensor has been demonstrated previously. There have been numerous strategies for enzyme immobilized electrodes for improving direct electron transfer (DET) pathways and control of non-specific adsorption, including site-mutagenesis [37] and a combination of strategies of self-assembled monolayer and deglycosylation [38]. In this study, the simple, low-cost, and reagent-free enzyme immobilization approach was used for electrode modification. Noble silver nanoparticles were studied and applied to develop biosensors because of their unique properties that include excellent electrical conductivity, easy preparation, and good catalytic activity [39]. In particular, the formal potential in converting Ag to AgCl or AgCl to Ag is in the middle of redox potentials of biological redox systems [35,40]. Herein, a two-step enzymatic reaction, glucose oxidase (GOx), and HRP, together with specific antibodies, are involved in this RFID wireless biosensor. Glucose oxidase enzyme-labeled cotinine antibodies are the vital biorecognition part for cotinine sensing. The conjugate also translates an immune reaction into a change of electrochemical and electromagnetic signals on the NFC-tag-based biosensor. In the analytical procedure, different levels of cotinine (0, 100, 200, 400, and 1000 ng/mL) in a PBS medium were separately tested with an in-house fabricated lateral flow immunochromatographic test strip. In the competitive immunoassay, free cotinine in the sample compete with BSA-cotinine spotted on the test strip to bind with the GOx labeled cotinine antibody, as displayed in Figure 1D. After the immunochromatography, the BSA-cotinine part of the nitrocellulose membrane was cut and placed onto the AgNP/HRP/AuNP-modified SPE and connected to the RFID tag, and the reaction was initiated after applying 5 mM of glucose substrate. In the first enzymatic reaction, GOx catalyzes the glucose oxidation and produces H_2_O_2_, causing the conversion of electrically conductive Ag to non-conductive AgCl in the second enzymatic reaction, which is catalyzed by HRP. These reactions occur on the completely assembled platform of the modified SPE/the cut nitrocellulose membrane/the assistant pad.

**Figure 3:**
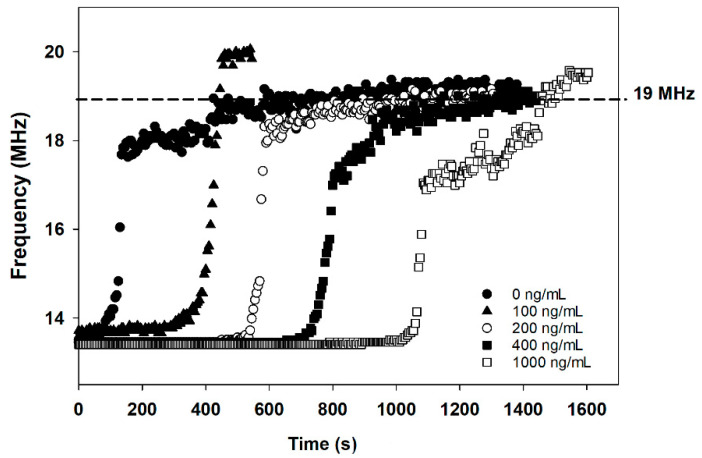
The resonance frequency (frequency at the lowest reflection of electromagnetic radiation from the tag) of NFC-tag-based immunosensor vs. time.

The frequency values at which the tag-based immunosensor shows the lowest reflection were monitored and collected at four-second intervals by the network analyzer. The frequency vs. time plots obtained with the tag-based immunosensor after being exposed to 5 mM of glucose are shown in Figure 3. The frequency-time traces pattern was remarkably similar, with a rapid frequency shift that occurred between 15 MHz and nearly 18 MHz. However, the time spent at the frequency of 13.56 MHz before reaching 18 MHz depends on cotinine concentrations. At lower concentrations, or in the absence of cotinine, the total duration before the frequency shift is shorter than the higher concentrations of cotinine. This can be explained as a consequence of the following reactions. Without cotinine in the sample, the higher amount of GOx-labeled cotinine antibody binds to the BSA-cotinine immobilized nitrocellulose membrane. The GOx-bound paper strip produces a higher concentration of H_2_O_2_, resulting in the rapid, HRP/AuNP assisted oxidation of Ag to AgCl on the electrode. The unconjugated GOx on the assistant pad was also used to accelerate the conversion of Ag to AgCl. On the other hand, with cotinine present in the analysis sample, this analyte binds to the GOx-conjugated cotinine antibody and results in occupied antibody binding sites. So, the antigen-antibody complex cannot be formed at the BSA-cotinine immobilized region. A low or absence of GOx bound to the cut nitrocellulose membrane leads to a low concentration of H_2_O_2_ and results in the prolonged oxidation of Ag on the SPE. The control experiments were elucidated by omitting the assistant pad in the supplementary information. Figure S1A,B reveal the amperometry results of PBS and 1000 ng/mL cotinine by the proposed method with and without GOx immobilized on an assistant pad. The decrease in current of PBS reaction was 23.7 µA (Figure S1B), whereas the decrease in current for 1000 ng/mL cotinine reaction was 12 µA. This observation suggests that the decrease in current for PBS was ~2 fold higher than cotinine containing PBS medium. By employing the assistant pad in the reaction, a 100 fold-enhancement in the rate of decrease in current was observed compared to the reaction without an assistant pad.

Besides the dramatic change in the electromagnetic and electrochemical signals, a change in the modified electrode’s microscopic appearance can be observed (see the Supplementary Materials). Field emission scanning electron microscope (FESEM) photographs of the modified SPE before cotinine assay at a magnification of 50,000× (Figure S2A) and 100,000× (Figure S2B) show the arrangement of AgNPs on the SPE. Figure S2C,D show the SPE after cotinine assay at the magnification of 50,000× and 100,000×, respectively. The aggregated particles of Ag/AgCl on the modified electrode (Figure S2C,D) resulted in the high resistivity of the modified SPE. In particular, crystals of NaCl were noticed on the modified electrodes after the enzyme-catalyzed Ag oxidation. Whereas the AuNPs on the electrode before (Figure S2E) and after cotinine assay (Figure S2F) are not changed in appearance that much when compared to AgNPs Furthermore, AFM imaging of the electrode was investigated and topographical 2D and 3D images were scanned over 5 µm × 5 µm area. The topographical images before (Figure S3A,B) and after (Figure S3C,D) cotinine determination revealed an increase in the height of the particle-decorated electrode and a gradual change in surface appearance.

**Figure 4:**
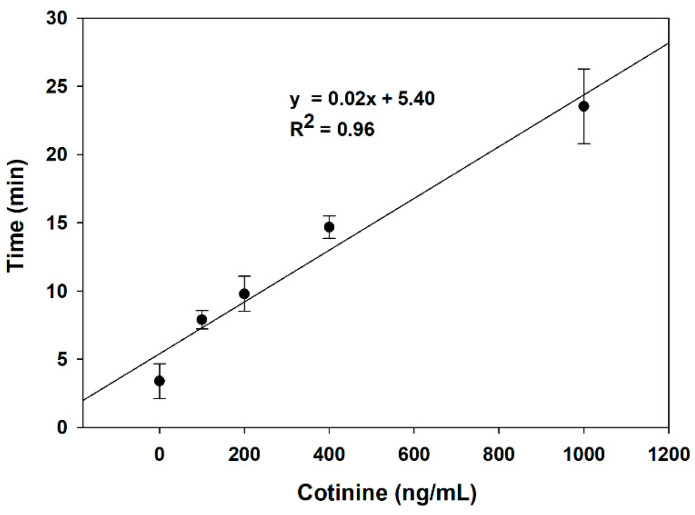
The relationship between cotinine concentration and time spent on Ag oxidation on the surface of the AgNP/HRP/AuNP-modified SPE.

The frequency change has occurred after 5 mM of glucose was supplied for generating H_2_O_2_ by the GOx-labeled cotinine antibody attached to the BSA-cotinine modified surface. The differences in frequency shift vs. time are due to the different oxidation rates of AgNPs to AgCl resulting from different amounts of GOx-labeled cotinine antibody attached to the BSA-cotinine modified surface at different cotinine concentrations. Figure 4 demonstrates the relationship between the delay time of the resonance frequency shift and cotinine concentration from triplicate experiments in the range of 0–1000 ng/mL (R^2^ = 0.96). As the analyzed data show, the time spent on Ag-AgCl conversion in PBS without cotinine is almost seven times lower than the 1000 ng/mL of cotinine. So, the duration of charge transfer from AgNPs to the heme-containing enzyme is dependent on the concentration of cotinine in the sample. The detection limit of the developed method is 189.7 ng/mL.

Previous works deploying bio or synthetic receptors for cotinine sensing in different mediums (Table S1) reported wide analytical ranges of their detection techniques from millimolar down to picomolar. Although our proposed method’s detection limit is not as sensitive as compared to other previous related works, our approach is very promising for end-users engagement in terms of simplicity and a possibility of easier integration into IoT solutions.

To demonstrate the applicability of the proposed method for wireless analysis based on smartphone detection, an android smartphone (Samsung Galaxy S7, Samsung Electronics Co., Ltd., Suwon, Korea) was installed with a mobile app developed in-house to automatically display time for Ag oxidation on the described tag-based immunosensor. The results were based on the time required for Ag oxidation from conductive Ag to non-conductive AgCl. The RFID tag was connected to the SPE modified electrode and tested with either cotinine-positive or negative samples. The smartphone was used as an RFID reader to read the tag until the Ag on the SPE was completely oxidized. The results demonstrate that the time required for Ag oxidation, as displayed on the screen, reflected the amount of cotinine in the sample. The time spent for Ag oxidation in the cotinine-free sample was 3 min and 54 s, while the cotinine-positive sample took 25 min and 2 s. The results are shown in the supplementary information (Video S1). Hence, we can confirm the possibility of a smartphone approach to personalized sensing of cotinine. However, the mobile app should be further improved to become more user-friendly with more informative display data.

### 3.1. Studies on Sample Matrix Effects toward Reaction on the Proposed Biosensor

To study the sample matrix effect on the rate of the Ag/AgCl reaction on the tag, real-time monitoring of the transformation of Ag into AgCl was conducted by electrical measurement of the current (amperometry) with an applied voltage of 5 mV between the short-circuited working and counter electrodes (the electrode configuration is shown in Figure 2B). AgNP/HRP/AuNP-modified SPEs were tested with different sample matrixes, including PBS and commercial control urine with cotinine levels of 0, 100, 200, 400, and 1000 ng/mL. The current flows through the silver bridge that electrically connected the working and counter electrodes of the SPE was expected to decrease to 0 A after the AgNPs were fully converted to the non-conductive state of AgCl. This amperometric measurement provides exclusive data on the change of current throughout the experiment.

The decreased current per unit of time (s) was calculated according to the equation:

(1) $$\frac{I\left(\mathrm{initial}\right)-I\left(\mathrm{final}\right)}{\mathrm{time}}$$

where *I* (initial) is the initial current (mA), *I* (final) is the final current in the reaction (mA), and time is the total time spending in the reaction (s).

**Figure 5:**
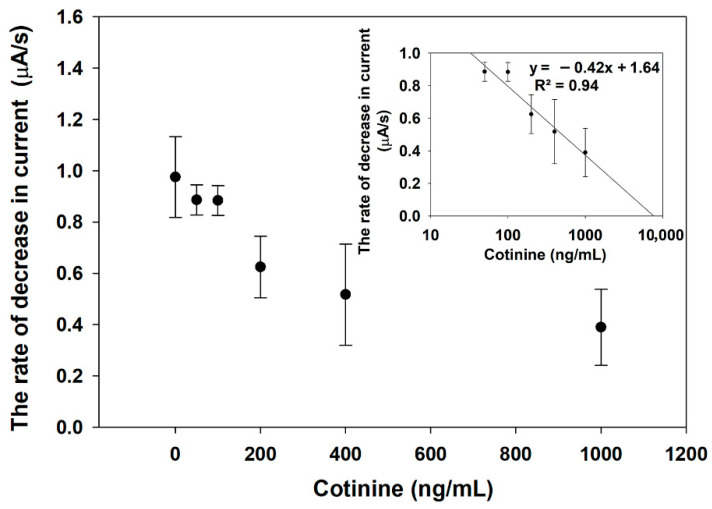
The dependence of a sensor response on the cotinine concentration in the PBS medium. The sensor response was measured as a rate of current decrease through an Ag layer connecting two electrodes on SPE.

In the PBS medium, the Ag-AgCl conversion rate was reciprocally dependent on the amount of cotinine, as shown in Figure 5. This may indicate that the conversion rates of Ag to AgCl at each cotinine concentration were the consequence of different quantities of H_2_O_2_. The amount of H_2_O_2_ generated at low levels or with no cotinine provides a faster Ag-AgCl conversion on the SPE, which can be monitored by amperometry. In contrast to the higher levels of cotinine, lower concentrations of produced H_2_O_2_ delay the time for the change on the decrease current. This is because of the competitive binding of cotinine in the sample to the GOx-labeled cotinine antibody. Lower levels or an absence of the unoccupied antibody can form the antibody-BSA-cotinine complex on the paper-based lateral flow strip. After glucose substrates were added to the system, the by-product H_2_O_2_ in the latter reaction was catalyzed by HRP on the modified electrode. The relationship between log cotinine concentration and the rate of decrease current was established as y = −0.42x + 1.64 (R^2^ = 0.94).

**Figure 6:**
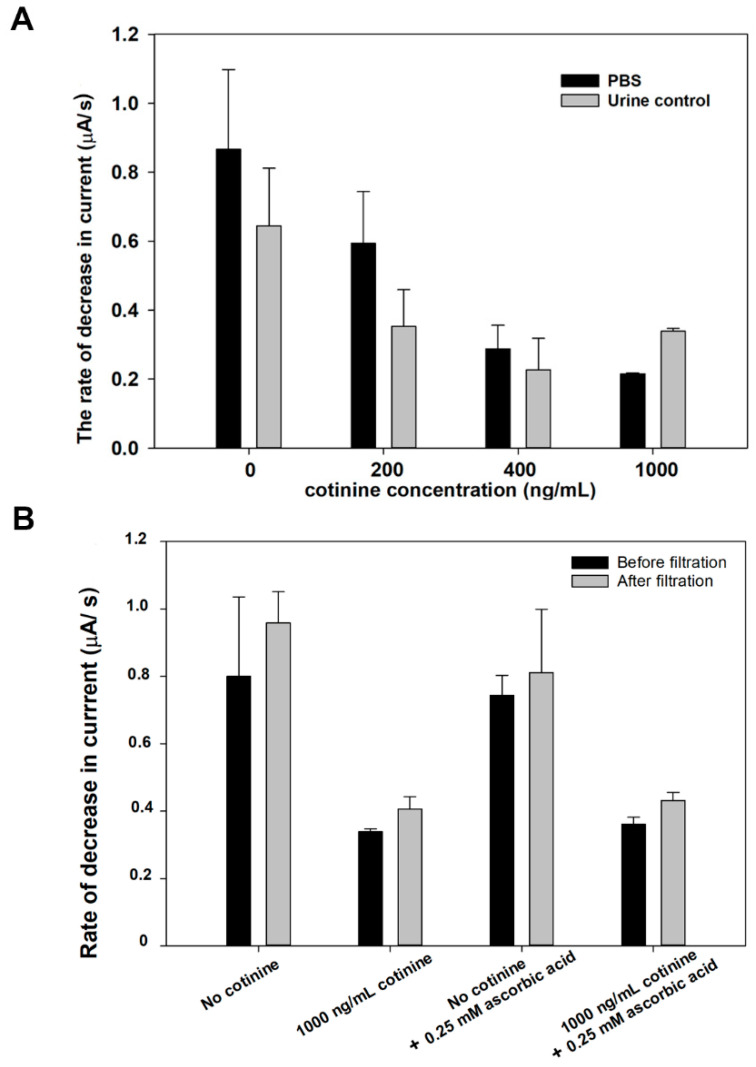
The conversion rates of Ag to AgCl in the PBS and urine control medium at different concentrations of cotinine (**A**). Rate of decrease of current through the layer of AgNPs on SPE when the immunosensor was exposed to different cotinine containing samples before and after sample processing with 0.5% Nafion/PTFE syringe filters (**B**).

From Figure 6A, the comparison between the rate of reaction occurring in the PBS and urine control was systematically studied. The obtained results from the amperometric measurement conducted in the PBS medium (solid bar) were higher than in the control urine (white bar) at cotinine concentrations ranging from 0 to 400 ng/mL. However, at 1000 ng/mL of cotinine, the decrease in current rate in the control urine is higher than that in the control urine with cotinine at 400 ng/mL. This may be the result of interfering substances in the samples that interfere with the antigen-antibody complex forming on the nitrocellulose membrane or other reactions constituting the sensor transduction mechanism. The control urine matrix contains several substances, including urea, uric acid, and creatinine, that might interfere with the activity of the enzymes and result in the lower conversion rate of Ag to AgCl when compared to cotinine in the PBS.

It is well-documented that many substances contained in a biological sample matrix, including ascorbic acid and uric acid, act as interferences in electrochemical measurement [41]. This challenge has led many research groups to try to find a way to suppress these interferences’ effect and increase the sensitivity of the detection method, such as by applying a selectively permeable membrane [42] or a special supported material for covering or immobilization of enzyme [43]. The bi-enzyme system is one of many strategies used to overcome the drawbacks and increase the selectivity of H_2_O_2_ measurement [44].

Research has shown the efficacy of Nafion in biosensing applications to protect the interfering anions [45]. However, directly applying Nafion on an electrode surface may block the electron shuttle [46]; this allows us to use the Nafion modified filter to process the sample before the cotinine assay. Figure 6B shows the rate of decrease in the current of the control urine matrix in each experiment. Although there was no statistical difference between the sampling process before and after filtration by paired sample *t*-test (*p* < 0.05) in all groups, the results revealed an increase in the Ag-oxidation rate after filtration with the Nafion modified membrane of approximately 9–20%. This could be due to the Nafion membrane exhibits the ability to reduce the matrix effect by negative charge repulsion. Interference molecules, including ascorbic acid and uric acid, can be trapped to the membrane due to the proton conductivity of Nafion.

### 3.2. Real Sample Analysis

Besides the non-invasive nature of the urine collection method, the average concentrations of cotinine in urine are approximately four to six times higher than those in saliva or blood [47], so it can be considered as a sensitive sample matrix for trace amount determination of smoking exposure [48]. As the results provided by the Toxicology Laboratory Service show, the pH range of all unknown urine samples is between 5.7–6.7, Table 1. The creatinine levels range from 43 to 228 mg/dL, and specific gravity is between 1.008 and 1.026. From the analysis by GC/MS, the positive samples containing cotinine have levels ranging from 98.8 to 280 ng/mL and nicotine concentrations between 80.8 to 256 ng/mL. To exemplify the proposed cotinine determination, AgNP/HRP/AuNP-modified SPE was tested with each unknown urine sample and further monitored by a network analyzer and electrochemical instrument. For wireless biosensors, cotinine-containing samples (A, B, C, and G) required more delay time for Ag-oxidation (over 60 min), while the negative samples required a shorter time for Ag–AgCl conversion (16–21 min). After the same positive samples were diluted at a ratio of 1:8 and re-examined by the proposed method, the duration of frequency shifts from 13.56 MHz to over 17.5 MHz decreased to 20–37 min, depending on the initial concentration of cotinine. The peak of frequency at 13.56 MHz represents the Ag-accommodated electrode before cotinine assaying, whereas the shift of frequency peak to 19 MHz demonstrates the transition state of Ag to AgCl. In comparison with the commercial cotinine lateral flow chromatography strip test results, a faint color appeared on the test line of all diluted samples, resulting in an inconclusive answer, as demonstrated in Table 1. However, the results from the diluted positive samples A and G by the wireless-based biosensor provided an Ag-oxidation time equivalent to the negative samples. This method can discriminate cotinine levels higher than 12 ng/mL, which means it is more sensitive than conventional immunochromatography. We also increased the amount of unconjugated glucose oxidase by two folds to accelerate the reaction, resulting in quicker Ag oxidation, as shown in Table S2.

**Figure 7:**
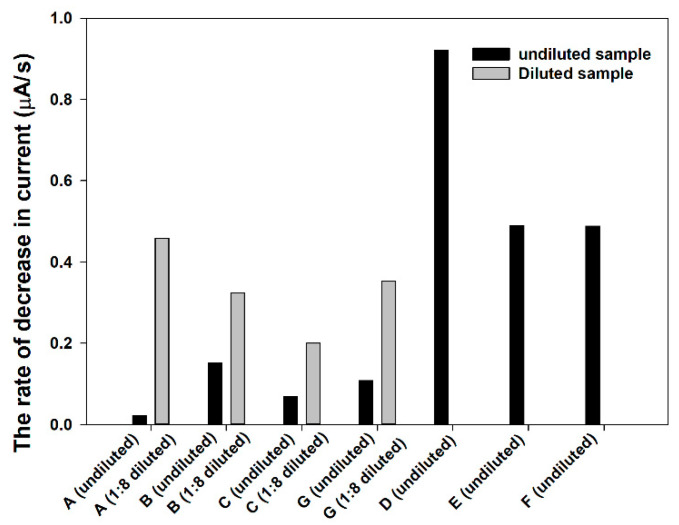
The conversion rates of Ag to AgCl in unknown urine samples.

From electrochemical measurement, the rate of decrease in current in the cotinine negative samples is higher than 0.4 µA/s, while the undiluted positive samples provide a value lower than 0.2 µA/s. When the target analytes in the positive samples were ascertained by testing of the diluted sample at the ratio of 1:8, the results (gray bar) showed an increase of Ag-oxidation rates (Figure 7). Besides the sample matrix aspect, the amount of silver nanoparticles, which is deposited on SPE, is another important optimized parameter that should be systematically optimized. This is due to the fact that the sensitivity of the proposed wireless biosensor depends on the amount of charge needed to transfer Ag to AgCl by the reactions catalyzed by the enzyme. Highly reproducible and low amount deposition of AgNPs will obviously enhance the sensitivity of sensors. Besides AgNPs, the combination of AgNPs and other nanomaterials, such as MXenes, graphene, or even conducting polymers, can be exploited in the proposed biosensor platform. Nevertheless, these materials must be systemically studied and investigated in future research

## 4. Conclusions

A simple lateral flow competitive immunochromatography was successfully integrated with the AgNP/HRP/AuNP-modified electrode and enabled for RFID sensing of cotinine in urine samples. The reaction between the cotinine and cotinine antibody in the sample was translated to the conversion of Ag to AgCl, resulting in the change of electromagnetic signal of the RFID tag. The time needed to accomplish this change was wirelessly monitored and was regarded as a biosensor signal response. Furthermore, the effect of different sample matrixes, including PBS, urine control material, and a simple sample manipulation process, was also investigated. The analyzed results demonstrated the potential of developing this wireless biosensor platform for real urine samples. The applicability of the RFID-based biosensor integrated with a simple paper-based immunochromatography for cotinine sensing was demonstrated as an easy-to-interpret platform that promises further development of immunosensors.

## Figures and Tables

**Table 1:** Urine analysis and nicotine metabolite determination by immunochromatography and gas chromatography, mass spectrophotometry, and the proposed wireless biosensor.

Name of Urine Unknown	Immunochromatography		Wireless Biosensor * (min)		GC/MS	
	Undiluted Sample	1:8 Diluted Sample	Undiluted Sample	1:8 Diluted Sample	Cotinine (ng/mL)	Nicotine (ng/mL)
A	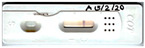 Positive	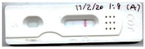 Negative	>60	20	98.8	80.3
B	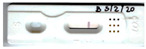 Positive	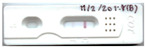 Negative	>60	35	280	148
C	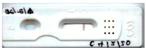 Positive	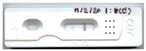 Negative	>60	37	270	256
D	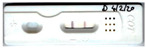 Negative	n.d.	21	n.d.	Not detected; <2	Not detected; <
E	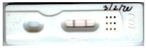 Negative	n.d.	20	n.d.	Not detected; <2	Not detected; <2
F	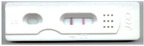 Negative	n.d.	16	n.d.	Not detected; <2	Not detected; <2
G	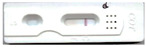 Positive	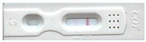 Negative	>60	21	128	140

* An assistant pad used in this experiment was immobilized with 19.29 mUGOx. n.d = not determined.

## Data Availability

Data is contained within the article.

## Supplementary Materials

The following are available online at https://www.mdpi.com/1424-8220/21/5/1659/s1, Figure S1: The amperometry of the proposed method with and without the assistant pad (**A**); the magnified scale of the graph of amperometric current versus time of the proposed method without the assistant pad (the inset) (**B**). Figure S2: FESEM micrographs of the AgNP/AuNP/HRP-modified SPE: AgNPs decorated area of SPE before cotinine assay at the magnification of 50,000× (**A**) and 100,000× (**B**) and after cotinine assaying at the magnification of 50,000× (**C**) and 100,000× (**D**). The AuNPs decorated area of SPE before cotinine assay (**E**) and after cotinine assay (**F**). Figure S3: Atomic force microscope images of the Ag-modified electrode. The 2D image (**A**) and 3D image (**B**) represent the modified SPE before cotinine determination. The 2D image (**C**) and 3D image (**D**) represent the modified SPE after cotinine assaying by the proposed method. Table S1: The recent reports summarizing of detection limit, detection range, media, and the setting for end-user. Table S2: Cotinine determination by a wireless-based biosensor. Video S1: Demonstration of the proposed cotinine assay by the wireless biosensor.
