# Supplementary material for: Paper-Based Competitive Immunochromatography Coupled with an Enzyme-Modified Electrode to Enable the Wireless Monitoring and Electrochemical Sensing of Cotinine in Urine
Source: Sensors (Basel). 2021 Feb 28;21(5):1659. doi: 10.3390/s21051659 (PMC7957614; doi:10.3390/s21051659)
Supplement: Supplementary file 1 [file sensors-21-01659-s001.zip › supplement/sensors-1116571-supplementary.pdf]

Supplementary Materials

Paper-Based Competitive Immunochromatography Coupled with an Enzyme-Modified Electrode to Enable the Wireless Monitoring and Electrochemical Sensing of Cotinine in Urine

Nutcha Larpant [1], Pramod K. Kalambate [1], Tautgirdas Ruzgas [2,3] and Wanida Laiwattanapaisal [1,4,*]

[1] Biosensors and Bioanalytical Technology for Cells and Innovative Testing Device Research Unit, Department of Clinical Chemistry, Faculty of Allied Health Sciences, Chulalongkorn University, Bangkok 10330, Thailand; Nutchalp.l@gmail.com (N.L.); Pramodkalambate1@gmail.com (P.K.K.)

[2] Department of Biomedical Science, Faculty of Health and Society, SE-205 06 Malmö, Sweden; Tautgirdas.ruzgas@mau.se

[3] Biofilms—Research Center for Biointerfaces, Malmö University, SE-205 06, Malmö, Sweden

[4] Department of Clinical Chemistry, Faculty of Allied Health Sciences, Chulalongkorn University, Bangkok 10330, Thailand

* Correspondence: Wanida.L@chula.ac.th

1. The Amperometry of the Proposed Method with and Without the Assistant Pad

Figure S1 reveals amperometric measurements with and without the assistant pad, and the inset shows the decrease in current in the reaction without the assistant pad. After the amperometry was conducted by applying the potential at 5 mV, all data was collected. The current of the reactions with the assistant pad decreased to 0 A within 2500 s. The fastest Ag-oxidation rate was obtained from reaction without cotinine which spent 2 fold shorter time than the reaction with cotinine. While omitting the GOx on the assistant pad experiment, the Ag-AgCl conversion rate in the reaction with cotinine was almost 2 times slower than the reaction without cotinine.

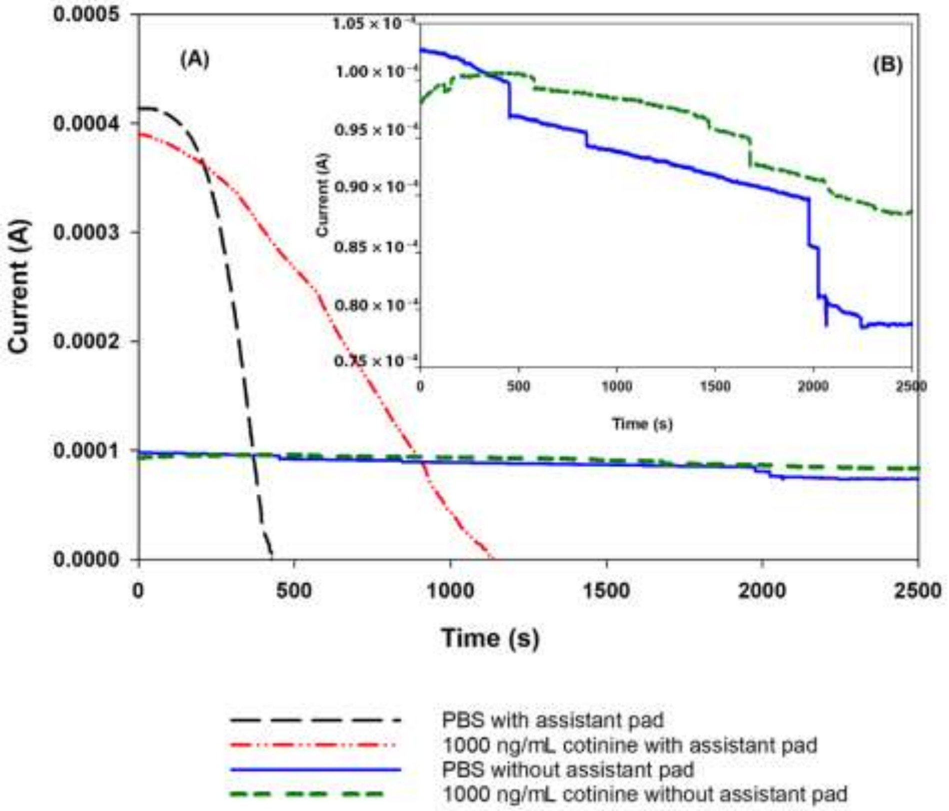

Figure S1. The amperometry of the proposed method with and without the assistant pad (**A**); the magnified scale of the graph of amperometric current versus time of the proposed method without the assistant pad (the inset) (**B**).

2. Morphological Analysis of Modified Electrodes

2.1. Field Emission Scanning Electron Microscope (FESEM)

FESEM was employed to investigate the AgNP/HRP/AuNP-modified electrodes' morphological changes before and after the cotinine assay. The micrographs of Ag-accommodated electrodes were recorded under the accelerating voltage of 5 kV and magnifications of 50,000x and 100,000x. As mentioned in the results and discussion section, there are gradual changes in the Ag-modified SPE's microscopic appearances after the Ag-oxidation reaction that occurs in the proposed cotinine assay. NaCl crystals can be noticed in Figure S2C,D. The AuNPs decorated area of SPE before cotinine assay (S2E) and after cotinine assay (S2F) are not changed in appearance that much when compared to AgNPs.

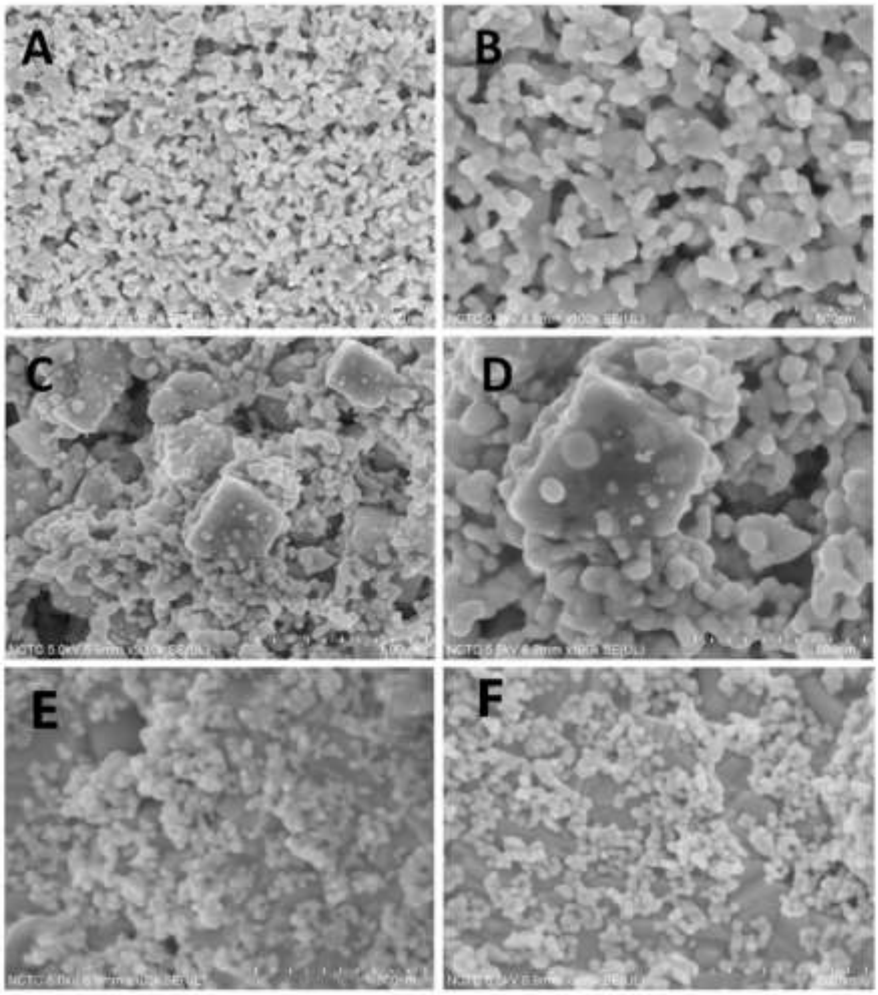

Figure S2. FESEM micrographs of the AgNP/AuNP/HRP-modified SPE: AgNPs decorated area of SPE before cotinine assay at the magnification of 50,000× (**A**) and 100,000× (**B**) and after cotinine assaying at the magnification of 50,000× (**C**) and 100,000× (**D**). The AuNPs decorated area of SPE before cotinine assay (**E**) and after cotinine assay (**F**).

2.2. Atomic Force Microscope

An atomic force microscope was also used to demonstrate the vertical change of the modified electrodes by taping mode over a dimension of 5 μm, obtaining two- and three-dimensional images. The 2D (Figure S3A) and 3D (Figure S3B) images of the electrode's surfaces were different from the appearance of the Ag-modified SPE after cotinine determination (Figure S3C,D) which is in accordance with the results of the SEM images.

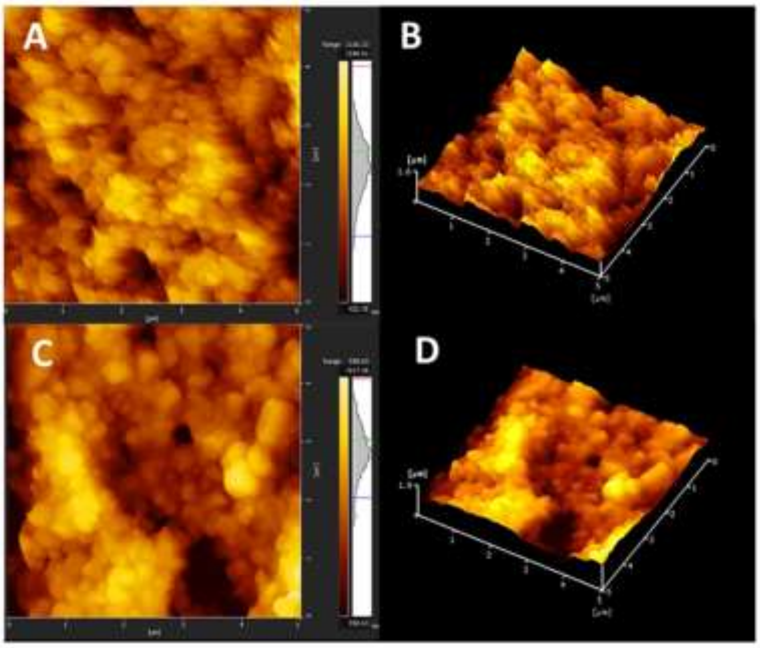

Figure S3. Atomic force microscope images of the Ag-modified electrode. The 2D image (**A**) and 3D image (**B**) represent the modified SPE before cotinine determination. The 2D image (**C**) and 3D image (**D**) represent the modified SPE after cotinine assaying by the proposed method.

3. Comparison of the Performance of the Recent Reports of Cotinine Detection

Table S1. The recent reports summarizing of detection limit, detection range, media, and the setting for end-user.

Detection techniques	Linear sensing range	Detection limit	Sample	Setting for end-user	Ref
Immunochromatography coupled with electrochemical detection	5.67–567 nM	5.67 nM	Serum	Electrochemical analyzer	[1]
MIP using electrochemical detection	1–100 nM	0.33 nM	Saliva	Electrochemical analyzer	[2]
Immunosensor coupled with electrochemical detection	1×10^{-1} to 1×10^{4} pg/ml	0.34 pM	Saliva	Electrochemical analyzer	[3]
Molecularly Imprinted Polymer-Carbon Nanotube based impedimetric sensor	-	0.57 μM	Cotinine in electrolyte	Electrochemical analyzer	[4]
Molecularly Imprinted Polymer-Carbon Nanotube based impedimetric sensor	-	0.27 mM	Cotinine in electrolyte	Electrochemical analyzer	[5]
Immunochromatography coupled with electrochemical detection and wireless sensing	1.13–5.67 μM	1.07 μM	Urine	Electrochemical analyzer or smart phone	This work

Table S2. Cotinine determination by a wireless-based biosensor.

Unknown Sample	Duration of the Cotinine Assaying on the Proposed Wireless Biosensor (min)	
	Undiluted	1:8 Diluted Sample
A	n.d.	18.3
B	n.d.	24.3
C	n.d.	30.5
D	15.3	n.d.
E	16.8	n.d.
F	13.7	n.d.
G	42	19.3

* An assistant pad used in this experiment was immobilized with 38.58 mUGOx. n.d. = not determined.

References

1. Nian, H.; Wang, J.; Wu, H.; Lo, J.-G.; Chiu, K.-H.; Pounds, J.G.; Lin, Y. Electrochemical immunoassay of cotinine in serum based on nanoparticle probe and immunochromatographic strip. *Anal. Chim. Acta.* **2012**, *713*, 50–155.
2. Parate, K.; Karunakaran, C.; Claussen, J.C. Electrochemical cotinine sensing with a molecularly imprinted polymer on a graphene-platinum nanoparticle modified carbon electrode towards cigarette smoke exposure monitoring. *Sens. Actuators B* **2019**, *287*, 165–172.
3. Lee, K.; Yoon, T.; Yang, H.; Cha, S.; Cheon, Y.-P.; Kashefi-Kheyrabadi, L.; Jung, H.-I. All-in-one platform for salivary cotinine detection integrated with a microfluidic channel and an electrochemical biosensor. *Lab Chip* **2020**, *20*, 320–331.
4. Abbas, Y.; Bomer, J.; Brusse-Keizer, M.; Movig, K.; van der Valk, P.; Pieterse, M.; Segerink, L.; Olthuis, W.; van der Berg, A. Imprinted Polymer-carbon Nanotube based Cotinine Sensor. *Procedia Eng.* **2016**, *168*, 497–500.
5. Antwi-Boampong, S.; Mani, K.S.; Carlan, J.; BelBruno, J.J. A selective molecularly imprinted polymer-carbon nanotube sensor for cotinine sensing. *J. Mol. Recognit.* **2013**, *27*, 57–63.
